# Extradural cervical spinal schwannoma in a child: a case report and review of the literature

**DOI:** 10.1186/s13256-019-2108-6

**Published:** 2019-07-17

**Authors:** Apar Pokharel, T. S. Rao, Prabhat Basnet, Bikash Pandey, Naganawalachulu Jayaprakash Mayya, Suvekshya Jaiswal

**Affiliations:** 1 Department of Otorhinolaryngology and Head and Neck Surgery, College of Medical Sciences, Chitwan, Nepal; 2 Department of Pathology, College of Medical Sciences, Chitwan, Nepal; 3 Department of Radiodiagnosis, College of Medical Sciences, Chitwan, Nepal

**Keywords:** Spinal schwannoma, Extradural schwannoma, Magnetic resonance imaging

## Abstract

**Introduction:**

Extradural schwannoma arising from high cervical spinal root is a rare entity in children. We report a case of extradural cervical schwannoma in a 14-year-old boy.

**Case presentation:**

Our patient is a 14-year-old Madhesi boy presenting with swelling in the posterior triangle of his neck. The radiological features suggested solitary extradural cervical schwannoma which was confirmed later by histopathological findings. There were no postoperative neurological complications in our patient.

**Conclusion:**

Extradural spinal schwannoma is a benign tumor. Gross total resection with good clinical outcome can be achieved with minimal risks.

## Introduction

The incidence rate of spinal tumors is 1.1 per 100,000 population [1]. Spinal cord tumors are a relatively rare diagnosis and account for 1 to 10% of all pediatric central nervous system tumors [2–4]. A large percentage of developmental tumors like dermoid, epidermoid, and teratomas are common in this population. Spinal schwannoma is relatively rare in the pediatric population [2–5].

Schwannomas arise from upper cervical spinal roots more commonly than from any other spinal nerves [6–8]. Approximately 75% of schwannomas are intradural, 10% intra-extradural, and the rest (15%) are extradural [9].

Case reports represent a study design to advance medical scientific knowledge especially of rare diseases. A solitary extradural cervical schwannoma is a very rare entity. There are no case reports of solitary extradural cervical spinal schwannomas in the pediatric population reported in the literature. In this article, we report the clinical and radiological features, surgical approach and findings, postoperative follow-up, and ultimate neurological outcome of high cervical extradural spinal schwannoma in a 14-year-old patient.

## Case presentation

**Fig. 1:**
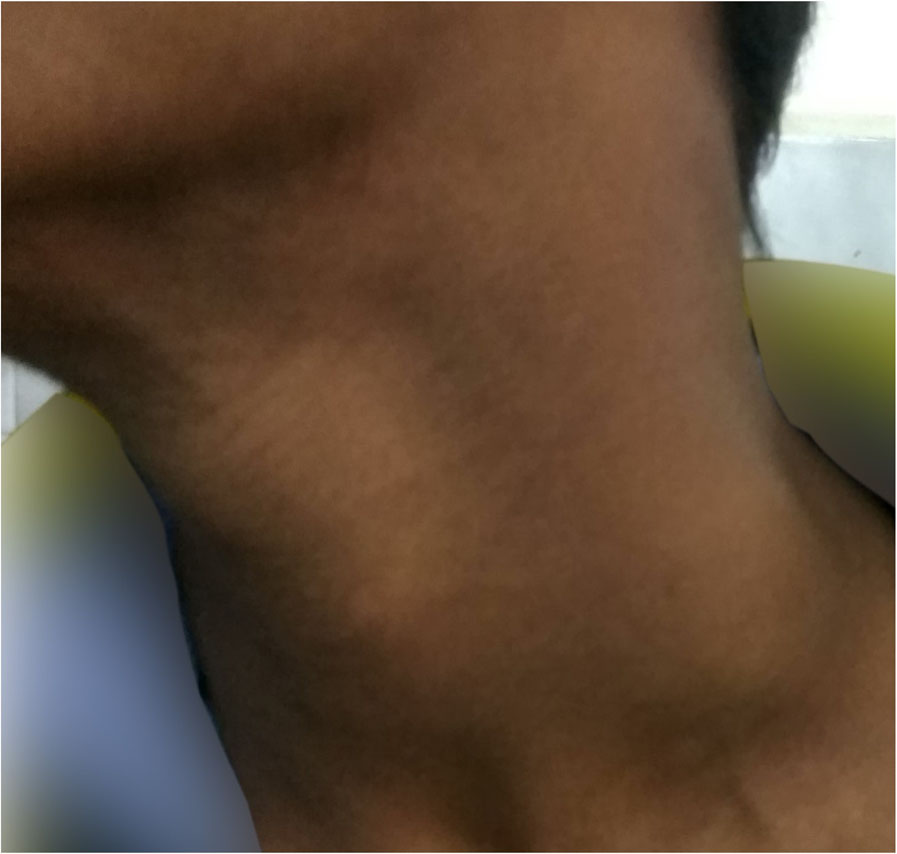
Swelling in the left posterior triangle of the neck

A 14-year-old Madhesi boy presented with a swelling in the left side of his neck for 2 years (Fig. 1). The swelling was insidious in onset and gradually increasing in size. There was no significant past medical or surgical history. He did not smoke tobacco. He was a student. He was not on any medications. There was no family history of any genetic diseases or malignancy.

**Fig. 2:**
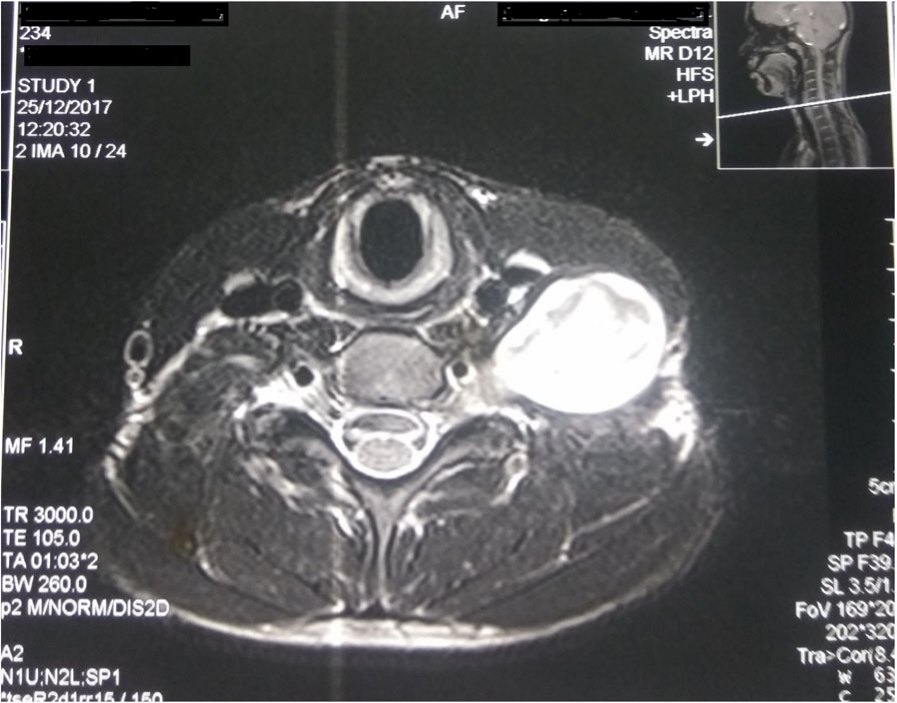
T2-weighted magnetic resonance imaging showing well-defined hyperintense mass in left posterior triangle extending up to neural foramina

On examination, a single swelling of approximately 5 cm × 4 cm in size was located in the posterior triangle on the left side of his neck. The swelling was mobile, non-tender, and firm in consistency. The rest of the head and neck examination was normal. On neurological examination, he had normal muscle strength, no sensory deficits, no pathological reflexes, and no long tract signs. Magnetic resonance imaging showed a well-defined T1-weighted isointense and T2-weighted hyperintense ovoid cystic mass with enhancing solid component within, measuring 6 × 4.5 × 2.5 cm involving the left posterior cervical space with extension into left C5–C6 neural foramina causing its widening. The mass was displacing the carotid space anteromedially. It was also abutting the posterior border of left sternocleidomastoid muscle causing its displacement anteriorly. Inferiorly, the mass extended into the left supraclavicular region (Fig. 2). His vital sign were stable. He was afebrile, pulse was regular and 80/minute, blood pressure was 110/80 mmHg, and respiratory rate was 14/minute.

No other clinical or radiological features of neurofibromatosis such as light brown spots on the skin, freckles in the armpit and groin, small bumps within nerves (neuromas), scoliosis, hearing loss, cataracts at a young age, balance problems, flesh-colored skin flaps, muscle wasting, vestibular schwannoma, meningioma, glioma, neurofibroma, or posterior subcapsular lenticular opacities were seen.

**Fig. 3:**
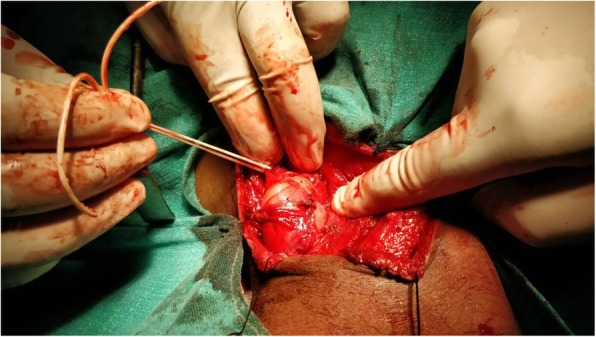
Dissection of schwannoma through supraclavicular incision

**Fig. 4:**
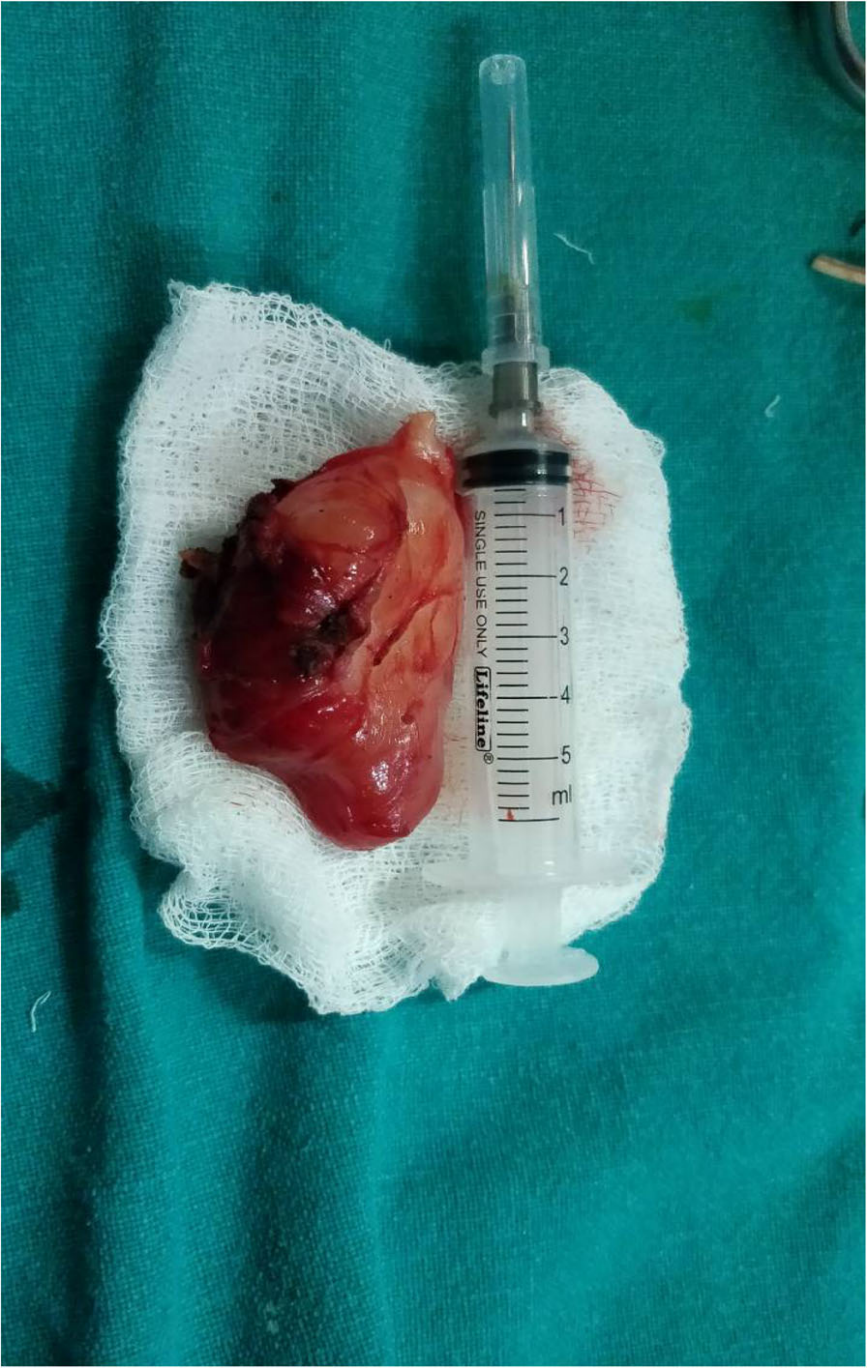
Surgical specimen

**Fig. 5:**
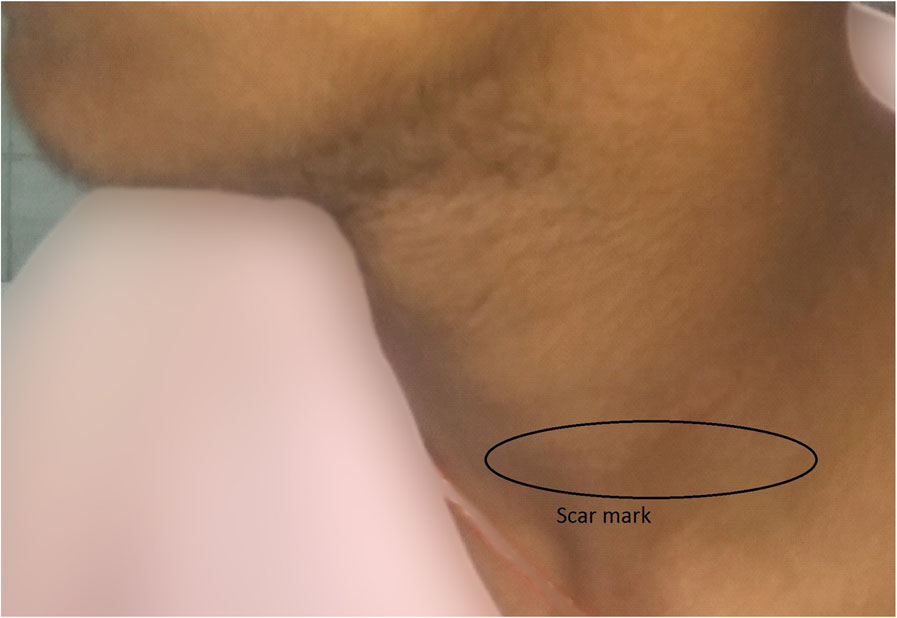
Follow-up of the patient after 1 year

The total leukocyte count and peripheral blood smear were normal. Urinary catecholamines level was normal. Fine-needle aspiration cytology of the swelling was done. The findings were consistent with schwannoma. An excision of our patient’s schwannoma was planned. A supraclavicular incision was made and subplatysmal flaps were elevated both superiorly and inferiorly. The sternocleidomastoid muscle was retracted medially and the tumor was excised completely. Although this extradural tumor extended through C5–C6 foramina, a laminectomy was not needed. The tumor was pulled out through the foramina (Figs. 3 and 4). His postoperative course was uneventful and he was discharged on sixth postoperative day without any change of neurological status. Neither any neurological complications nor any recurrence were seen in our patient after 1 year of follow-up (Fig. 5).

**Fig. 6:**
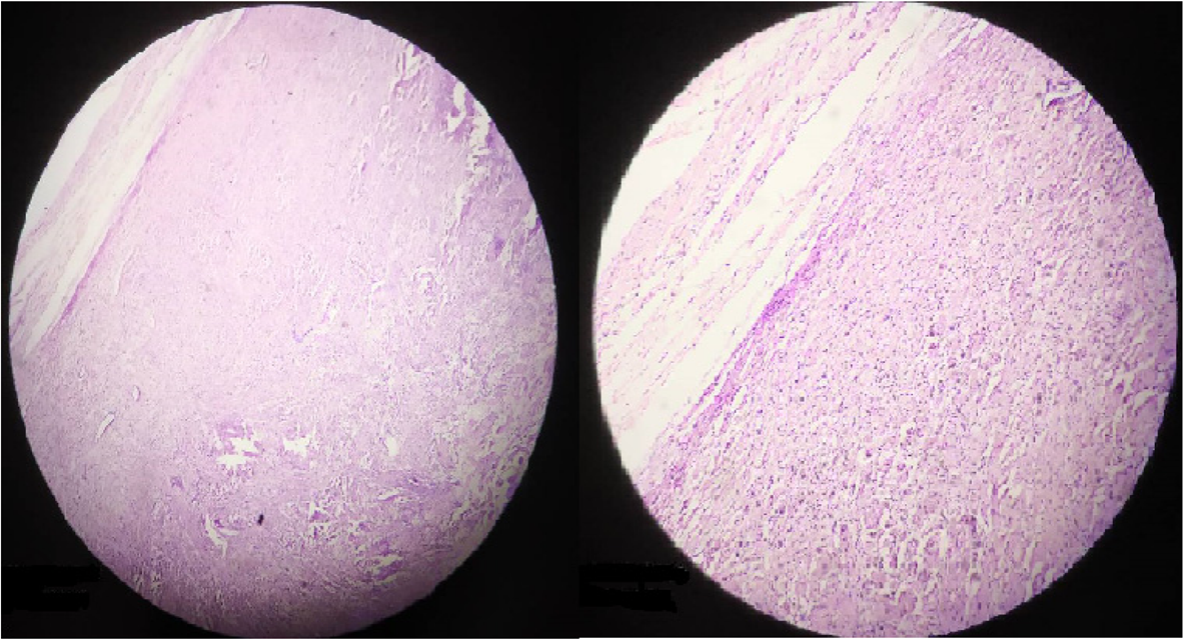
Histopathological findings showing a well-capsulated lesion showing hypocellular and hypercellular areas respectively

Histopathological findings were consistent with the diagnosis of schwannoma. Microscopic examination of the specimen revealed tissue surrounded by fibrous capsule. The tumor tissue showed predominantly cellular (Antoni A) admixed with hypocellular areas (Antoni B). Antoni A areas consisted of compact spindle cells having prominent nuclei. The nuclei were arranged in a palisading pattern (Fig. 6).

## Discussion

Solitary extradural cervical spinal schwannoma is a rare disease. This case report describes the case history and management of cervical spinal schwannoma. The treatment was surgical excision and the patient had no neurological deficit after surgery.

The most common spinal cord tumors are intramedullary [5]. Based on anatomic location, the tumors can be divided into three discrete areas. Extradural tumors are present between the bony structures and the dura. Next are intradural tumors, which are subdivided into extramedullary and intramedullary. Extramedullary tumors are present within the dura but not part of the spinal cord whereas intramedullary tumors are within the spinal cord parenchyma [10]. Dumbbell-shaped cervical schwannoma involves both the spinal canal and the posterior triangle of the neck. Dumbbell tumors have both intraspinal and paraspinal components which are connected through a frequently enlarged and eroded intervertebral foramen. Most dumbbell-shaped schwannomas are located in the thoracic spine. Although the extraspinal component of schwannoma is usually larger than the intraspinal tumor part, the intraspinal tumor is responsible for typical symptoms like local pain and symptoms from spinal cord compression in the cervical spine [11].

In published series of spinal nerve sheath schwannoma, extradural tumors have not been systematically addressed and they are only mentioned in association with more frequent intradural and intradural–extradural tumors. The disease is more prevalent in the fifth decade of life and more common in females. Extradural cervical spinal cord schwannoma in the pediatric population has never been reported in the literature [12, 13].

The differential diagnosis of spinal masses includes neurogenic tumors (schwannoma, neurofibroma) and meningiomas [11]. Approximately 30% of all primary spinal cord tumors constitute spinal schwannomas. Around 70% of spinal schwannomas arise from sensory roots, 20% from motor roots, and the other 10% arise from both motor and sensory roots [14, 15]. Among the spinal schwannomas arising from C1, C2, and C3 spinal roots, the most common tumor arises from C2 spinal root. It constitutes 15% of all spinal schwannomas [6–8].

Multiple schwannomas are seen in type 2 neurofibromatosis. Multiple schwannomas are seen in 4% of cases in the spinal cord [16].

Symptoms and signs of this condition are usually diagnostic in nature. These include radicular pain, swelling in the neck, and motor weakness. These are slow growing tumors and usually attain a large size before becoming symptomatic [7]. Sometimes the tumor can present with unusual symptoms like syncopal attacks, migraine headaches, and unrelated motor and sensory symptoms. This can lead to misdiagnosis [7, 17]. Our case involved a young boy with no motor and sensory symptoms except for a firm swelling in the posterior triangle in his neck.

Schwannomas are moderately vascular tumors and are firm in consistency [7].

On histopathologic examination, spinal schwannomas have Antoni A and Antoni B patterns. Type A tissue is cellular and demonstrates nuclear palisading and Verocay bodies. Verocay bodies represent prominent extracellular matrix and secretion of laminin. Antoni type B is a loosely organized tissue with myxomatous and cystic changes and may also represent degenerated Antoni A tissue [18]. They are relatively simple to resect. For dissection, they have a well-defined arachnoid plane intradurally and well-defined capsule extradurally. For rapid and sustained neurological recovery, total tumor resection is advocated [7]. Recurrence has been reported in a partially excised tumor. Every attempt should be made to remove the tumor completely [7, 8]. In our case, the extradural tumor was resected completely and no recurrence was seen even after 1 year of follow-up.

## Conclusion

Extradural cervical spinal schwannoma in the pediatric population is a rare encounter. These are benign tumors. Gross total resection of the tumor can be achieved with minimal risk and a good clinical outcome.
